# *In Vitro* Schistosomicidal Activity of the Alkaloid-Rich Fraction from *Ruta graveolens* L. (Rutaceae) and Its Characterization by UPLC-QTOF-MS

**DOI:** 10.1155/2019/7909137

**Published:** 2019-11-16

**Authors:** Lara Soares Aleixo de Carvalho, Lucas Sales Queiroz, Ismael José Alves Junior, Ayla das Chagas Almeida, Elaine Soares Coimbra, Priscila de Faria Pinto, Marcos Paulo Nascimento da Silva, Josué De Moraes, Ademar A. Da Silva Filho

**Affiliations:** ^1^Faculty of Pharmacy, Department of Pharmaceutical Sciences, Federal University of Juiz de Fora, Juiz de Fora, MG 36036-900, Brazil; ^2^Department of Parasitology, Microbiology and Immunology, Biological Sciences Institute, Federal University of Juiz de Fora, Juiz de Fora, MG 36036-900, Brazil; ^3^Department of Biochemistry, Biological Sciences Institute, Federal University of Juiz de Fora, Juiz de Fora, MG 36036-900, Brazil; ^4^Núcleo de Pesquisa em Doenças Negligenciadas, Universidade de Guarulhos, Guarulhos, SP 07025-000, Brazil

## Abstract

Schistosomiasis is a neglected tropical disease that affects million people worldwide, mostly in developing countries. *Ruta graveolens* (Rutaceae) is a plant used in folk medicine to treat several diseases, including parasitic infections. In this study, we reported the *in vitro* schistosomicidal activity of the *R. graveolens* extract (**Rg**) and its active fraction (**Rg-FAE**). Also, the characterization of **Rg-FAE** by UPLC-ESI-QTOF-MS analysis and its *in vitro* antileishmanial activity against *Leishmania braziliensis* were also performed. *In vitro* schistosomicidal assays were assessed against adult worms of *S. mansoni*, while cell viability against peritoneal macrophages was measured by MTT assay. **Rg** (100 *μ*g/mL) exhibited noticeable schistosomicidal activity, causing 100% mortality and decreasing motor activity of all adult male and female schistosomes, but with low activity against *L. braziliensis*. After chromatographic fractionation of **Rg**, fraction **Rg-FAE** was obtained, showing high activity against adult schistosomes. UPLC-ESI-QTOF-MS analysis of **Rg-FAE** revealed the presence of eleven alkaloids and one furanocoumarin. No significant antileishmanial activity was found for **Rg**, while **Rg-FAE** exhibited activity against *L. braziliensis* promastigotes. We demonstrated, for the first time, that the *R. graveolens* extract (**Rg**) and its alkaloid-rich fraction (**Rg-FAE**) are active against adult worms of *S. mansoni*, with no significant cytotoxicity on macrophages. Our findings open the route to further antiparasitic studies with the active fraction of *R. graveolens* and its identified compounds, especially alkaloids.

## 1. Introduction

Schistosomiasis is a neglected tropical disease (NTD) caused by *Schistosoma* parasites, mainly *S. mansoni*, that is associated with long-term undernutrition, anaemia, organ scarring, and fibrosis, resulting in disabling patient symptoms [1]. About 190 million people are infected worldwide with *Schistosoma* infections, with more than 70 million of new cases and thousands of deaths annually registered [2]. Only in Brazil, around 8 million people are infected with this chronic debilitating disease [3]. However, the treatment of schistosomiasis is based on only one drug, praziquantel (PZQ), which has a limited effect on already developed liver and spleen lesions [4].

Leishmaniasis, also a NTD, is caused by the protozoan *Leishmania* and transmitted by infected female phlebotomine sand flies. Leishmaniasis is endemic in more than 95 countries of tropical and subtropical areas, with more than 1 million of cases worldwide every year [5]. Although some antileishmanial compounds have been registered as medications, such as amphotericin B, pentamidine, and miltefosine, none of the available drugs can be considered perfect because of their high toxicity, long duration of treatment, and severe adverse reactions, which often lead to treatment abandonment [5]. In this scenario, there is an urgent need for new and better antileishmanial drugs [5, 6].

In this regard, *Ruta graveolens* (Rutaceae), also known as “rue,” has been used in the folklore medicine for the treatment of several inflammatory diseases, such as rheumatism [7], and also to treat cutaneous leishmaniasis [8, 9] in Brazil. Previous studies showed that *R. graveolens* exhibits antiparasitic activity against *Leishmania amazonensis* [9] and contains several biologically active metabolites, such as alkaloids and coumarins [8, 10]. Meanwhile, neither schistosomicidal studies nor antileishmanial activities against *Leishmania braziliensis* has not yet been described to *R. graveolens*.

Thus, the aim of this study was to evaluate the *in vitro* schistosomicidal activities of the hydroalcoholic extract and the alkaloid-rich fraction from *R. graveolens*. Also, the characterization of the alkaloid-rich fraction from *R. graveolens* by UPLC-ESI-QTOF-MS analysis and its *in vitro* antileishmanial activity against *Leishmania braziliensis* were also performed.

## 2. Materials and Methods

### 2.1. Plant Material and Extraction

Aerial parts of *R. graveolens* L. were collected at the Faculty of Pharmacy's Medicinal Herb Garden, Juiz de Fora city, MG, Brazil, in January, 2017. A voucher specimen (CESJ 70472) was identified and stored at the Herbarium of the Botany Department of the Federal University of Juiz de Fora, MG, Brazil.

Plant material (250 g) was dried, powdered, and exhaustively extracted by maceration at room temperature, using EtOH : H_2_O (8 : 2 v/v). After filtration, the solvent was removed under reduced pressure to yield 25 g of the crude hydroalcoholic extract of *R. graveolens* (**Rg**). The crude extract of *R. graveolens* (**Rg**) (22 g) was chromatographed over silica gel (70–230 mesh, Merck) using a vacuum liquid chromatography system (VLC, glass columns with 5–10 cm i.d) and hexane-ethyl acetate mixtures in increasing proportions as eluents, furnishing 4 fractions: **Rg-FC1** (960 mg), **Rg-FC2** (220 mg), **Rg-FC3** (670 mg), and **Rg-FAE** (1700 mg). Based on its schistosomicidal and antileishmanial activities, fraction **Rg-FAE** was selected for UPLC-ESI-QTOF-MS analysis.

### 2.2. UPLC-ESI-QTOF-MS Analysis

#### 2.2.1. LC Conditions

The ultraperformance liquid chromatograph (UPLC) analysis was carried out, using an Acquity UPLC system (Waters Corporation, Milford, MA, USA) equipped with a binary pump, inline degasser, and autosampler coupled to an electrospray ionization quadrupole time-of-flight tandem mass spectrometer (ESI-Q-TOF/MS) (Waters Corporation, USA). Separation was carried out on BEH C_18_ column (100 mm × 2.1 mm, 1.7 *μ*m, Milford, USA). The mobile phase consisted of LC grade water with 0.1% formic acid (A) and LC grade acetonitrile (B) with the following gradient profiles: 0–2 min, 5% B; 2–14 min, 5–98% B; 14–16 min, 98% B; and 16–20 min, 98–5% B. The flow rate was 0.4 mL·min^−1^. Before the analysis, samples were dissolved in methanol (10 mg·mL^−1^), centrifuged at 10,000 rpm, filtered using a 0.22 *μ*m filter, and injected (injection volume of 15 *μ*L).

#### 2.2.2. MS Conditions

Mass spectrometry was performed with a XEVO G2S QTOF mass spectrometer (Waters Corporation, Milford, MA, USA) with ESI operating in the positive ion mode for scanning. The scanning range was *m/z* 150–1200. The capillary voltage was 2.5 kV, the low collision energy was 6 eV, and the higher collision energy was 15–30 eV. The ion source temperature was 120°C, and the desolvation temperature was 450°C. Nitrogen was used as the source of desolvation gas (800 L·h^−1^) and cone gas (50 L·h^−1^). For accurate mass measurements, data were centroided during acquisition, and 200 pg·mL^−1^ of leucine-enkephalin (*m/z* 565.2771) (Sigma-Aldrich, Steinheim, Germany), dissolved in acetonitrile/0.1% formic acid (50 : 50, v/v), was infused continuously as an external reference (LockSpray™) into the ESI source with automatic mass correction enabled. The data were processed using Chromalynx™ application manager with MassLynx™ 4.1 software (Waters Corporation, Milford, MA, USA). Besides the observed MS spectra and data obtained by QTOF-MS analysis, the main tools for compound identification were the interpretation of the observed QTOF-MS spectra in comparison with those found in the literature and several online databases (ChemSpider, MassBank, and Spectral Database for Organic Compounds).

### 2.3. Schistosomicidal Assays

#### 2.3.1. Parasite

*Schistosoma mansoni* (BH strain) worms were maintained in *Biomphalaria glabrata* snails as intermediate hosts and *Mesocricetus auratus* hamsters as definitive host at the Adolfo Lutz Institute (São Paulo, Brazil), according to standard procedures previously described [11]. At 49 days after infection, adult *S. mansoni* specimens were recovered from each hamster by perfusion in the Roswell Park Memorial Institute (RPMI) 1640 medium (Invitrogen, So Paulo, Brazil) and supplemented with heparin. All experiments were authorized by the Committee for Ethics in Animal Care of Adolfo Lutz Institute (São Paulo, Brazil), in accordance with nationally and internationally accepted principles for laboratory animal use and care (CEUA ≠11.794/08). The study was conducted in adherence to the institution's guidelines for animal husbandry.

#### 2.3.2. *In Vitro* Studies with *S. mansoni*

Adult schistosomes were washed in the RPMI 1640 medium (Gibco) and supplemented with 200 *μ*g/mL streptomycin, 200 IU/mL penicillin (Invitrogen), and 25 mM Hepes. Adult worm pairs (male and female) were incubated in a 24-well culture plate (Techno Plastic Products, TPP, St. Louis, MO, USA), containing the same medium supplemented with 10% heat-inactivated calf serum (Gibco BRL) at 37°C in a 5% CO_2_ atmosphere. For the *in vitro* test with *S. mansoni*, a preliminary screening of the crude extract (**Rg**) and its fractions **Rg-FC1**, **Rg-FC2**, **Rg-FC3**, and **Rg-FAE** were evaluated at 100 *μ*g/mL, according to previously described [12]. The most active sample (**Rg-FAE**) was also evaluated at lower concentrations (3.125 to 50 *μ*g/mL). Samples were added to the culture from a 4000 *μ*g/mL stock solution in RPMI 1640, containing dimethyl sulfoxide (DMSO). The final volume in each well was 2 mL. The control worms were assayed in the RPMI 1640 medium, and RPMI 1640 with 0.5% DMSO as control group and PZQ (2 *μ*M) was used as the reference drug. All experiments were performed in triplicate and were repeated at least two times. Parasites were maintained for 72 h and monitored every 24 h using a light microscope in order to evaluate their general conditions, such as motor activity and mortality rate [13].

### 2.4. Antileishmanial Assays

#### 2.4.1. Parasite Culture

Promastigotes of *L. braziliensis* (MHOM/Br/75/M2903) were cultivated in the BHI medium (Himedia, Mumbai, India) supplemented with 10% inactivated fetal bovine serum (FBS) (Cultilab, So Paulo, Brazil), L-glutamine, penicillin at 100 UI/mL, and streptomycin at 100 *μ*g/mL (Cultilab, So Paulo, Brazil) and kept in a BOD incubator at 25°C.

#### 2.4.2. *In Vitro* Antileishmanial Activities

Promastigotes of *L. braziliensis*, at 2 × 10^6^ cells/mL, were incubated with different concentrations (3.125 to 50.0 *μ*g/mL) of the *R. graveolens* crude extract (**Rg**) or its alkaloid-rich fraction (**Rg-FAE**) for 72 h at 25°C, according to previously described [14]. Parasite viability was evaluated by MTT assay, and percentages of the inhibition growth were expressed in comparison with untreated control. For the intracellular amastigote assays, peritoneal macrophages, obtained from BALB/c mice, were added in the RPMI 1640 medium (Cultilab, So Paulo, Brazil) supplemented with 10% FBS at 2 × 10^6^ cells/mL. Adherent macrophages were infected with *L. braziliensis* promastigotes in the stationary growth phase (MOI = 10) and incubated for 4 h in 5% CO_2_ at 33°C. After washing, various concentrations (6.25 to 50.0 *μ*g/mL) of the *R. graveolens* crude extract (**Rg**) or its alkaloid-rich fraction (**Rg-FAE**) were added for 72 h, according to previously described [14]. The slides were stained with Giemsa, and the number of amastigotes was determined using light microscopy. The results were expressed in percentage of inhibition of the number of amastigotes, compared with untreated control. All procedures were performed in agreement with the Ethical Principles in Animal Research and according to protocols approved by the Ethical Committee for Animal Research (CEUA≠012/2015).

### 2.5. Cytotoxicity Assay

Peritoneal macrophages obtained from BALB/c mice were treated with different concentrations (4.69 at 75.0 *μ*g/mL) of the *R. graveolens* crude extract (**Rg**) and its alkaloid-rich fraction (**Rg-FAE**) for 72h, according to previously described [14]. Results were determined by MTT assay, and all procedures were performed in agreement with the Ethical Principles in Animal Research and according to protocols approved by the Ethical Committee for Animal Research (CEUA ≠013/2015).

### 2.6. Statistical Analysis

Statistical tests were performed with the Graphpad Prism (version 4.0) software. Significant differences were determined by one-way analysis of variance (ANOVA) and applying Tukey's test for multiple comparisons with a level of significance set at *P* < 0.05.

## 3. Results and Discussion

The demand for new therapeutic alternatives against the 20 groups of the so-called NTDs is a worldwide need since the few drugs available are often associated with severe side effects and high toxicity [1, 6, 15]. In this context, plant-derived natural products constitute a quite important starting point for new therapies or for the development of new drugs against NTDs, due to their vast chemical diversity and already known antiparasitic potential [15].

Considering the promising antiparasitic potential of Rutaceae species, in this work, we have highlighted the antischistosomal activity of an alkaloid-rich fraction from the *R. graveolens* extract. To our knowledge, this is the first report for the schistosomicidal activity of *R. graveolens* against adult worms of *S. mansoni.* Also, we have evaluated the antileishmanial activity of *R. graveolens* against *L. braziliensis*, which has not been documented in the literature.

First, the survival and motor activities of *S. mansoni* adult worms, after *in vitro* incubation with the crude extract of *R. graveolens* (**Rg**), were analyzed. As shown in Table 1, **Rg** (100 *μ*g/mL) exhibited noticeable schistosomicidal activity, causing 100% mortality and decrease of motor activity of all adult male and female schistosomes (Table 1).

Schistosomicidal activities have been reported for several extracts from Rutaceae species or their secondary metabolites, mainly for alkaloids and coumarins [16–19]. In this regard, ethanolic extracts of *Zanthoxylum naranjillo* (Rutaceae) showed a significant activity on egg reduction of adult schistosomes [16], while ethanolic extracts of *Citrus reticulata* (Rutaceae) roots showed significant *in vivo* schistosomicidal activity [17]. Also, the alkaloid epiisopiloturine, isolated from the leaves of *Pilocarpus microphyllus* (Rutaceae), showed an *in vitro* effect on schistosomula and adult worms of *S. mansoni*, with no apparent cytotoxicity on mammalian cells [18]. Other compounds, such as furanocoumarins from the leaves of *Citrus* species (Rutaceae), have also been evaluated for their schistosomicidal activity [19].

After, **Rg** was chromatographed into four fractions, which were also assayed against schistosomes. In the schistosomicidal assay, when tested at 100 *μ*g/mL, only the fraction **Rg-FAE** was active (Table 1), causing 100% mortality and decreasing motor activity after 24 hours of incubation, while fractions **Rg-FC1**, **Rg-FC2**, and **Rg-FC3** did not show any activity for adult schistosomes, even at the highest concentration tested (100 *μ*g/mL) (Table 1). When analyzed at lower concentrations, **Rg-FAE** showed a pronounced schistosomicidal activity at 50, 25, 12.5, and 6.25 *μ*g/mL, causing significant decrease in motor activity and death of all male adult worms (Table 1). In contrast, when adult worms were maintained in the RPMI medium containing 0.5% DMSO, their appearance was similar to those maintained in the same medium without DMSO even after 72 h of incubation. During this period, all parasites revealed normal motor activity with natural peristalsis of the worm body. PZQ (0.6248 *μ*g/mL or 2 *μ*M), used as the reference drug, reduced the motility and caused the death of all the parasites after 24 h of incubation.

Interestingly, at concentrations lower than 25 *μ*g/mL, marked schistosomicidal selectivity of **Rg-FAE** to male mortality was observed (Table 1). With respect to differential drug susceptibility between male and female schistosomes, several works have been reported showing that male worms of *S. mansoni* are often more susceptible than female worms [20–23]. Some compounds showed higher selectivity to male adult worms, such as *N*-alkylated diamines and amino alcohols [20], while preferential killing of females was reported to other drugs [21, 22], including artesunate [23]. The fact that male schistosomes were more susceptible to **Rg-FAE** raised the question of whether the observed effect could be due to tegumental damages of compounds presented in **Rg-FAE** since tegument is extremely important to the parasite surviving both *in vitro* and in the host [24].

**Figure 1:**
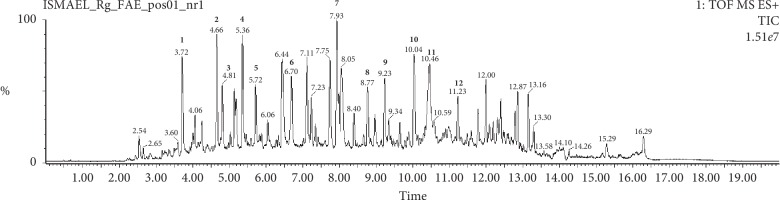
Typical UPLC-ESI-QTOF-MS chromatogram of *R. graveolens* fraction- (**Rg-FAE-**) positive mode.

**Figure 2:**
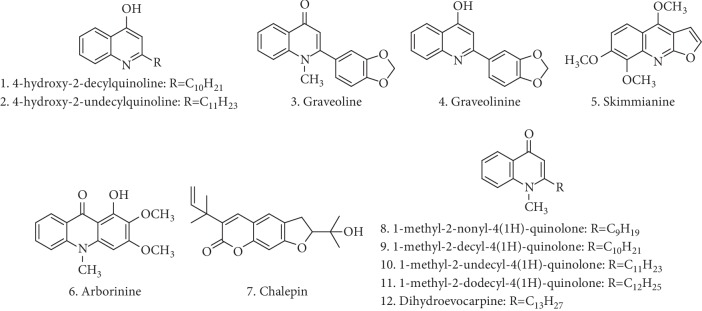
Chemical structures of compounds identified in **Rg-FAE** by UPLC-ESI-QTOF-MS analysis.

Considering the chemical characterization of the active fraction, qualitative chromatographic profiles of **Rg-FAE** were obtained by UPLC-ESI-QTOF-MS on the positive mode (Figure 1). The detailed information of each peak is listed on Table 2. Chemical structures of all identified compounds (Figure 2) in the active fraction (**Rg-FAE**) from *R. graveolens* were proposed through the interpretation of their mass spectra fragmentation patterns in comparison with those found in the literature and several online databases. A total of 11 alkaloids, along with one furanocoumarin, were identified on the basis of the contrasting cleavage rules, fragmentation ion pattern, and mass spectral data.

Mass data analysis showed that compounds **1** and **2** are quinoline alkaloids, presenting the same *m/z* fragmentation pattern in the positive ion mode (*m/z* 198, 188, 184, 172, and 132). Peak **1** (*m/z* 286.0753) was suggested as 4-hydroxy-2-decylquinoline (compound **1**, Figure 2) and peak **2** (*m/z* 300.0867) as 4-hydroxy-2-undecylquinoline (compound **2**, Figure 2) by comparing their mass spectra data with the literature [25].

Peaks **3** (4.81 min) and **4** (5.36 min) were isomers, showing the same molecular formula (C_17_H_13_NO_3_), but displaying different MS/MS patterns. It was observed that the parent ion-radical (*m/z* 280.0962) undergoes a loss of CH_3_, producing ion fragments at an *m/z* of 265.0717 [M-CH_3_]^+^. Peak **3** also showed a loss of CO, giving the *m/z* of 237.0768 [M-CH_3_-CO]^+^. As previously reported [26], the loss of CO, from the molecular ion-radical, may lead to the formation of the indole scaffold peak. Finally, a loss of formaldehyde may take the mass fragment of *m/z* 207.0654 [M-CH_2_O]^+^. Mass fragmentation data for peak **3** are in agreement with the proposed structure of graveoline (compound **3**, Figure 2) [27, 28]. Similarly, peak **4** showed a loss of an OCH_3_ methoxyl group, producing fragments at *m/z* of 250.0862 [M-OCH_3_]^+^, suggesting that compound **4** may be graveolinine (Figure 2) [27, 28].

Peak **5** (*t*_R_ = 5.72) showed an [M+H]^+^ ion at *m/z* 260.0886 and fragment ions at *m/z* 245.0661 [M-CH_3_]^+^ and 230.0430 [M-CH_3_]^+^ in MS^2^ mode, suggesting consecutive losses of 15 u, which may be due to the loss of methyl groups from methoxyl groups. In addition, fragment ions were observed at *m/z* 216 [M-CH_3_-CO]^+^ and 199 [M-CH_3_-H_2_O-CO]^+^. Molecular ion and fragmentation patterns are similar to those reported from literature [28, 29], indicating that compound **5** is skimmianine (Figure 2). Similarly, peak **6** (*t*_R_ = 6.70) was identified as arborinine (Figure 2) based on its positive molecular ion at [M + H] ^+^ of *m/z* 286.1064, as well as by MS/MS studies and fragmentation pattern of previous reports [27]. In addition, peak **7** (*t*_R_ = 7.93) showed a molecular ion [M + H]^+^ at *m/z* 315.1586 and an ion fragment [M-(CH_3_)_2_COH]^+^ at *m/z* 255. Based on its fragmentation pattern along with previous literature data [30], this compound was identified as furanocoumarin chalepin (compound **7**, Figure 2).

According to literature, the McLafferty rearrangement occurs in quinolone alkaloids, leading to the formation of stable conjugate systems with ion fragments at *m/z* 186 and *m/z* 173 [32]. Therefore, *m/z* 186 and 173 ion fragments were used as diagnostic ion fragments to identify the quinolone alkaloids **8**, **9**, **10**, **11**, and **12** (Figure 2), which differ only in the number of carbons of the side chain. Then, comparing the mass spectra data with the literature [31], peaks **8** (*m/z* 286.2171), **9** (*m/z* 300.2355), **10** (*m/z* 314.2481), **11** (*m/z* 328.2617), and **12** (*m/z* 342.2783) were identified, respectively, as quinolone alkaloids 1-methyl-2-nonyl-4(1H)-quinolone, 1-methyl-2-decyl-4(1H)-quinolone, 1-methyl-2-undecyl-4(1H)-quinolone, 1-methyl-2-dodecyl-4(1H)-quinolone, and dihydroevocarpine, respectively. All of these quinolone alkaloids (**8**, **9**, **10**, **11**, and **12**) were previously identified in *R. graveolens* [33].

In addition, the effect of the crude extract **Rg** was evaluated against *L. braziliensis*. However, no significant antileishmanial results were found for **Rg** (IC_50_ > 50 *μ*g/mL) against *L. braziliensis* promastigotes (data not shown). In contrast, previous antileishmanial study with a crude extract of *R. graveolens* against *L. amazonensis* showed an inhibition of 74.4% in the number of promastigotes at 100 μg/mL [9]. On the contrary, the alkaloid-rich fraction **Rg-FAE** exhibited pronounced activity against *L. braziliensis* promastigotes in the antileishmanial assay, inhibiting the parasites growth in all concentrations, displaying an IC_50_ value of 5.90 *μ*g/mL, which was better than the reference drug miltefosine (IC_50_ value of 12.09 ± 0.017 *μ*g/mL). However, **Rg-FAE** showed low activity against intracellular amastigotes of *L. braziliensis*, diminishing the number of intracellular amastigotes by 26.58% at the maximum concentration used (50 *μ*g/mL), while miltefosine showed an IC_50_ value of 2.95 ± 0.44 *μ*g/mL. This difference in sensibility between both stages of parasite could be due to biochemical targets, the rate of division, exposure, and inactivation into the parasitophorous vacuole or drug metabolism [34]. Although the antileishmanial effects of **Rg-FAE** cannot be considered as promising as well as the schistosomicidal activity, our data contribute with the ethnopharmacological use of a traditional medicinal plant from the Brazilian flora, such as *R. graveolens*, for the treatment of Leishmaniasis.

Moreover, considering their safety, **Rg** and **Rg-FAE** were also evaluated on cytotoxicity assay against murine macrophages. No significant toxic effects were observed for **Rg** (CC_50_ > 75 *μ*g/mL) or **Rg-FAE** (CC_50_ value > 75 *μ*g/mL) to mammalian cells (Table 1) at concentrations that effectively kills worms of *S. mansoni* and promastigotes of *L. braziliensis*, giving support to its potential in identifying lead compounds for the development of novel antiparasitic drugs.

*R*. *graveolens* is an important medicinal plant that has been used as anthelmintic and to treat several diseases, such as leishmaniasis [9, 10]. Alkaloids and coumarins, present in this plant species, have showed antileishmanial, antimalarial, and trypanocidal activities [35]. Among the compounds identified in **Rg-FAE**, several alkaloids, along with the identified furanocoumarin, could be related to the antiparasitic activity of this fraction.

Regarding the antiparasitic activity of **Rg-FAE** and its chemical composition, it was shown that 2-substituted quinoline alkaloids are highly active *in vitro* and *in vivo* against *Leishmania* sp. [36]. Also, some quinolone and quinoline alkaloids have showed some activity against larval [37] and adult worms [38], schistosomes. Since *R. graveolens* possesses a wide pharmacological potential and may have low toxicity [10], additional investigations are necessary to determine the antiparasitic potential of this species, especially of its active alkaloid-rich fraction **Rg-FAE** in treating schistosomiasis and leishmaniasis.

## 4. Conclusions

The present study has demonstrated, for the first time, that the *R. graveolens* extract and its alkaloid-rich fraction are active against adult worms of *S. mansoni in vitro*, with no cytotoxicity on mammalian cells. Eleven alkaloids, together with a furancoumarin, were identified by UPLC-ESI-QTOF-MS analysis as constituents of the active fraction Rg-FAE. Our findings open the route to further antiparasitic studies with the active fraction and its isolated compounds, especially alkaloids.

## Figures and Tables

**Table 1:** *In vitro* schistosomicidal and cytotoxic activities of the crude extract of *R. graveolens* (**Rg**) and its fractions (**Rg-FC1**, **Rg-FC2**, **Rg-FC3**, and **Rg-FAE**) against adult worms of *S. mansoni* incubated for 24 h.

Groups	Dead worms (%)^a^		Decrease of motor activity (%)^a^		Cytotoxicity CC_50_ (*μ*g/mL)^e^
	Male	Female	Male	Female	
Control^b^	0	0	0	0	—
0.5% DMSO	0	0	0	0	—
PZQ^c^	100	100	100	100	—
					
**Rg** ^d^	100	100	100	100	>75
					
**Rg-FC1** ^d^	0	0	0	0	—
					
**Rg-FC2** ^d^	0	0	0	0	—
					
**Rg-FC3** ^d^	0	0	0	0	—
					
**Rg-FAE**					
100 *μ*g/mL	100	100	100	100	>75
50 *μ*g/mL	100	100	100	100	—
25 *μ*g/mL	100	40	100	100	—
12.5 *μ*g/mL	100	0	100	100	—
6.25 *μ*g/mL	100	0	100	100	—
3.125 *μ*g/mL	0	0	0	0	—

^a^Percentages relative to 20 worms investigated; ^b^RPMI 1640; ^c^tested at a concentration of 2 *μ*M; ^d^tested at a concentration of 100 *μ*g/mL; ^e^CC_50_ values (50% cytotoxicity concentration) on peritoneal macrophages.

**Table 2:** Chemical characterization of **Rg-FAE** by UPLC-ESI-QTOF-MS.

Peak	Proposed compounds	RT (min)	*m/z* experimental [M+H]^+^	Main fragments via MS/MS	Molecular formula	References
**1**	4-hydroxy-2-decylquinoline	3.72	286.0753	198.0591, 188.0791, 184.0822, 172. 0781, 132.0473	C_19_H_27_NO	[25]
**2**	4-hydroxy-2-undecylquinoline	4.66	300.0867	198.0937, 188.0735, 184.0739, 172.0781	C_20_H_29_NO	[25]
**3**	Graveoline	4.81	280.0962	265.0717, 237.0768, 207.0654	C_17_H_13_NO_3_	[26, 27]
**4**	Graveolinine	5.36	280.0962	265.0717, 250.0862, 222.0900	C_17_H_13_NO_3_	[26, 27]
**5**	Skimmianine	5.72	260.0886	245.0661, 230.0430, 216.0645, 199.0618	C_14_H_13_NO_4_	[28, 29]
**6**	Arborinine	6.70	286.1064	271.0844, 253.0732, 244.1687, 225.0770, 197.0848, 182.0599	C_16_H_15_NO_4_	[27]
**7**	Chalepin	7.93	315.1586	273.1148, 259.1003, 255.1037, 241.0889, 223.0753, 213.0933, 201.0573	C_19_H_22_O_4_	[30]
**8**	1-methyl-2-nonyl-4(1H)-quinolone	8.77	286.2171	186.0907, 173.0827	C_19_H_27_NO	[31]
**9**	1-methyl-2-decyl-4(1H)-quinolone	9.23	300.2355	186.0907, 173.0827	C_20_H_29_NO	[31]
**10**	1-methyl-2-undecyl-4(1H)-quinolone	10.04	314.2481	186.0907, 173.0827	C_21_H_31_NO	[31]
**11**	1-methyl-2-dodecyl-4(1H)-quinolone	10.46	328.2617	186.0907, 173.0827	C_22_H_33_NO	[31]
**12**	Dihydroevocarpine	11.23	342.2783	186.0907, 173.0827	C_23_H_35_NO	[31]

## Data Availability

The data used to support the findings of this study are included within the article.
